# Genistein inhibits tumor invasion by suppressing multiple signal transduction pathways in human hepatocellular carcinoma cells

**DOI:** 10.1186/1472-6882-14-26

**Published:** 2014-01-16

**Authors:** Shulhn-Der Wang, Bor-Chyuan Chen, Shung-Te Kao, Ching-Ju Liu, Chia-Chou Yeh

**Affiliations:** 1 School of Post-Baccalaureate Chinese Medicine, Tzu Chi University, Hualien, Taiwan; 2 Department of Chinese Medicine, Buddhist Dalin Tzu Chi General Hospital, 2 Min-Sheng Road, Dalin Town, Chia-Yi 62247, Taiwan; 3 School of Chinese Medicine, China Medical University, Taichung, Taiwan; 4 Division of Chinese Medicine, China Medical University Hospital, Taichung, Taiwan; 5 School of Post-Baccalaureate Chinese Medicine, Collage of Chinese Medicine, China Medical University, Taichung, Taiwan

**Keywords:** Genistein, TPA, Matrix metalloproteinase 9, Tumor invasion, Nuclear factor-κB, Activator protein 1

## Abstract

**Background:**

Genistein (Gen) exhibits anti-mutagenic and anti-metastatic activities in hepatoma cell lines. Gen has suppressive effects on tumor growth and angiogenesis in nude mice. Gen suppresses the enzymatic activity of matrix metalloproteinase (MMP)-9; however, the mechanism underlying its anti-invasive activity on hepatocellular carcinoma (HCC) cells is unclear.

**Methods:**

In this study, the possible mechanisms underlying Gen-mediated reduction of 12-*O*-Tetradecanoylphorbol-13-acetate (TPA)-induced cell invasion and inhibition of secreted and cytosolic MMP-9 production in human hepatoma cells (HepG2, Huh-7, and HA22T) and murine embryonic liver cells (BNL CL2) were investigated.

**Results:**

Gen suppressed *MMP-9* transcription by inhibiting activator protein (AP)-1 and nuclear factor-κ B (NF-κB) activity. Gen suppressed TPA-induced AP-1 activity through inhibitory phosphorylation of extracellular signal-related kinase (ERK) and c-Jun N-terminal kinase (JNK) signaling pathways, and TPA-stimulated inhibition of NF-κB nuclear translocation through IκB inhibitory signaling pathways. Moreover, Gen suppressed TPA-induced activation of ERK/phosphatidylinositol 3-kinase/Akt upstream of NF-κB and AP-1.

**Conclusions:**

Gen and its inhibition of multiple signal transduction pathways can control the invasiveness and metastatic potential of HCC.

## Background

Hepatocellular carcinoma (HCC) is the fifth most common cancer and the third most frequent cause of cancer-related mortality worldwide, with 6,000,000 new cases diagnosed annually [1,2]. HCC is prevalent in Sub-Saharan Africa and Southeast Asia, including Taiwan. HCC is associated with various risk factors, including chronic infection with hepatitis B or C viruses, environmental carcinogens, chronic alcohol abuse, and non-alcoholic fatty liver disease [2,3]. HCC is a hypervascular tumor, commonly involving venous invasion, and HCC often progresses to intra- and extra-hepatic metastases [4]. Invasion and metastasis of cancer cells are the primary causes of cancer deaths, which are complicated processes involving proteolytic enzymes that participate in the degradation of environmental barriers such as the extracellular matrix and basement membrane. Among these enzymes, the matrix metalloproteinases (MMPs), which comprise a family of zinc-dependent endopeptidases, are intimately involved in the invasion and metastasis of various tumor cells [5,6]. The MMP family is involved in extracellular matrix degradation and is also associated with malignancy and metastasis. The *MMP-9* gene is strongly expressed in invasive HCC [7], and the MMP-9 protein content in HCC is greater than in the surrounding liver parenchyma. Therefore, MMP-9 may be used as a marker for the invasiveness and metastatic potential of HCC [7]. The activity of MMP-9 is tightly controlled, with regulation occurring primarily at the transcriptional level [5]. The *MMP-9* promoter is highly conserved and contains multiple functional elements, including nuclear factor-κB (NF-κB) and activator protein (AP)-1 elements [8-10].

12-*O*-Tetradecanoylphorbol-13-acetate (TPA) is one of the most widely used agents for studying the mechanisms of carcinogenesis [11]. TPA exhibits numerous biological effects by altering gene expression, a process that involves activation of protein kinase C (PKC) [12]. In addition to carcinogenesis, TPA induces MMP-9 expression via PKC-dependent activation of the Ras/extracellular signal-regulated protein kinase (ERK) signaling pathway, thus increasing the invasiveness of cell lines [13]. Previous reports have demonstrated that TPA-activated NF-κB and AP-1 activities, and increased MMP-9 expression in response to NF-κB activation, are associated with tumor metastasis [14].

Genistein (Gen; 5,7,4′-trihydroxyisoflavone), a soybean-derived isoflavone, has been identified as a potential cause for the low incidence of certain types of tumors, such as lung [15], breast, gastric, colon, and prostate cancers, and HCC [9,15-19]*.* Gen is also a principal chemopreventive component of soy and exerts a wide array of chemopreventive activities in each stage of multistep carcinogenesis [20,21]. Previous studies [20,22] showed that Gen is a promising agent for inhibiting the metastatic potential of HCC. Gen may affect HCC progression as a result of its effects on cell cycle progression and apoptosis [16,22]; however, there are no reports on the mechanism of the inhibitory effects of Gen on TPA-induced invasion and MMP-9 expression. Herein, we demonstrate that the suppression of TPA-induced MMP-9 activity by Gen occurs via disruption of NF-κB and AP-1 signaling pathways in HepG2 cells.

## Methods

### Reagents

Genistein (Sigma Chemical Co., St. Louis, MO, USA) was dissolved in 0.1 M Na_2_CO_3_ to create a 10-mM stock solution. TPA (Sigma-Aldrich, St. Louis, MO) was prepared in phosphate-buffered saline (PBS; 137 mM NaCl, 1.4 mM KH2PO4, 4.3 mM Na2HPO4, 2.7 mM KCl, pH 7.2). For analysis of the signaling pathways involved in TPA-induced DNA-binding of AP-1 and NF-κB, we also treated HepG2 cells with the p38 inhibitor SB203580 (SB), the MEK/ERK inhibitor PD98059 (PD), the JNK inhibitor JNKI, the IKK inhibitor BMS (AKTI), LY294002 (LY, an Akt inhibitor) and bisindolylmaleimide (GF, GF109203X, a PKC inhibitor) were purchased from Sigma-Aldrich to block these pathways.

### Cell culture and TPA treatment

Human hepatoma cell lines (HepG2, Huh-7, and HA22T) and murine embryonic liver cells (BNL CL2) were maintained in Dulbecco’s modified Eagle medium (DMEM; Life Technologies, Gaithersburg, MD, USA) and supplemented with 10% fetal bovine serum (FBS; HyClone, Logan, UT, USA). The cells were transiently transfected with 5 μg of plasmid DNA using SuperFect transfection reagent (Qiagen, Valencia, CA, USA). TPA (Sigma-Aldrich) was prepared in PBS (137 mM NaCl, 1.4 mM KH_2_PO_4_, 4.3 mM Na_2_HPO_4_, and 2.7 mM KCl, pH 7.2). HepG2, Huh-7, HA22T, and BNL CL2 cells were cultured in 25-cm^2^ flasks at 37°C. The flasks were immediately capped and sealed with parafilm to minimize evaporation. Cell growth was measured using a modified 3-(4,5-dimethylthiazol-2-yl)-2,5-diphenyltetrazolium bromide (MTT) assay. HepG2 cells were resuspended with 100 μL in 96-well plates and cultured with or without 80 μM TPA and Gen, incubated for 24 h, then 20 μL MTT was added to each well and incubated at 37°C for 4 h. The supernatant was removed, 200 μL dimethyl sulfoxide (DMSO) was added to each well to solubilize the formazan product, and the absorbance was measured at 470 nm using a microplate reader (Sigma).

### Wound healing assay

Hepatoma cell lines were grown to 90% confluence in a 6-well plate at 37ºC in a 5% CO_2_ incubator. A wound was created by scratching cells with a sterile 200 μL pipette tip, then the cells were washed twice with PBS to remove floating cells and added to serum-free medium. Photos of the wound were obtained via microscopy under 100× magnification.

### Invasion assay

Cell invasion was assessed using Matrigel-coated film inserts (8-μm pore size) fit into 24-well invasion chambers (Becton-Dickinson Bioscience, Franklin Lakes, NJ, USA). HepG2 cells (5 × 10^4^) were suspended in 200 μL of DMEM and added to the upper compartment of an invasion chamber in the presence or absence of 80 μM TPA; DMEM (500 μL) was added to the lower chamber. The chambers were incubated at 37ºC in a 5% CO_2_ atmosphere. The filter inserts were removed after a 24-h incubation period, and cells on the upper surfaces of the filters were removed with cotton swabs. Cells on the lower surfaces of the filters were stained with crystal violet, and the number of cells was determined with the use of a microscope. Final values were calculated as the mean of the total number of cells from 3 filters.

### Zymography

Gelatin zymography was used for determination of expression and activities of MMP-9 in TPA-treated (with or without Gen) human HepG2 cells. HepG2 cells were seeded in 100-mm plates using serum-free medium and pretreated with TPA and different concentrations of Gen. After incubation for 24 h, the conditioned media were collected and quantification of the protein concentrations was performed using the Bio-Rad protein assay (Bio-Rad Laboratories, Inc., Hercules, CA, USA). Culture supernatants were subjected to electrophoresis on gelatin substrate gels (10% sodium dodoecyl sulfate [SDS]-polyacrylamide gels containing 1 mg/mL gelatin). Subsequently, the gels were treated with 2.5% Triton X-100 for 30 min, followed by incubation for 24 h at 37°C in a buffer containing 100 mM Tris–HCl, pH 7.4, 0.15 M NaCl, and 15 mM CaCl_2_. The gels were stained with Coomassie Blue R-250 and then destained with water until emergence of clear zones that indicated proteolytic activity against a blue background.

### Luciferase assay

Wild-type sequences were obtained for *NF-κB* (GGAATTCCCC) and *AP-1* (TGAGTCA) sites. Reporter plasmids (pNF-κB-Luc and pAP-1-Luc) were purchased from Stratagene (La Jolla, CA, USA). Plasmid DNAs were prepared with a Qiagen Plasmid Midi Kit (Qiagen). The *MMP-9*-Luc plasmid was kindly provided by Dr. C.K. Glass [23]. Hepatoma cell lines were treated with 80 μM TPA for 8 h, and luciferase activity was determined as previously described [14]. Briefly, HepG2 cells in each well were washed with PBS and at lysed with 50 μL of passive lysis buffer (Promega, Madison, WI, USA) at various time points after treatment. Lysates were transferred to 96-well white plates and substrate was added (Promega) to assess the luciferase activity with a microplate reader (Synergy HT, Bio-Tek, Winooski, VT, USA). Relative luciferase activity was calculated by dividing relative luciferase units of *MMP-9*, *NF-κB*, or *AP-1* reporter plasmid-transfected cells by the relative luciferase units of pGL3-basic–transfected cells.

### Preparation of nuclear extracts and electrophoretic mobility shift assay

HepG2 cells were treated with 80 μM TPA and 10–40 μM Gen. Nuclear extracts were prepared as described previously [24]. Briefly, cells were stimulated, harvested by centrifugation, washed twice with cold PBS, and then nuclear extracts were prepared using NE-PER reagent (Pierce, Rockford, IL, USA), according to the manufacturer’s instructions. Biotin-labeled complementary oligonucleotides corresponding to *NF-κB* and *AP-1* binding sites were annealed. Biotinylated electrophoretic mobility shift assays (EMSAs) were performed as previously described [8], and gels were transferred to nylon membranes after electrophoresis. Membranes were blocked in solution and detected with alkaline phosphatase-conjugated streptavidin (Chemicon, Australia) followed by chemiluminescence (Roche, Germany).

### Western blot analysis

Hepatoma cells were treated with 80 μM TPA and 10–40 μM Gen and lysed in 250 μL of sample buffer (62.5 mM Tris–HCl, 2% SDS, 10% glycerol, 50 mM dithiothreitol, and 0.1% bromophenol blue, pH 6.8). We also collected the supernatants from cultures treated with 80 μM TPA and Gen. The supernatants were concentrated 40-fold with a Minicon filter (Millipore, Billerica, MA, USA) with a 15-kDa cutoff pore diameter. Protein concentrations were determined with a BCA Protein Assay Kit (Pierce, Rockford, IL, USA). To investigate the different cell fractions, the cells were scraped into 2 mL of buffer A (20 mmol/L Tris HCl, pH 7.5, 0.25 mol/L sucrose, 10 mmol/L EGTA, 2 mmol/L EDTA, 20 μg/mL leupeptin, 10 μg/mL aprotinin, and 200 μmol/L phenylsulfonyl fluoride) at 4°C and were sonicated and separated into cytosolic fraction and membrane fraction as described previously [25]. The cytoplasmic extracts (cytosol) were prepared using Cytoplasmic Extraction Reagent (Pierce), according to the manufacturer’s instructions. For translocation of p65, the protein concentrations of p65 in cytoplasmic extracts and nuclear extracts were detected by western blotting. Proteins (10 μg for cell lysates; 40 μg for supernatants) were separated using 10% SDS-polyacrylamide gel electrophoresis, and protein bands were transferred electrophoretically to nitrocellulose membranes. Membranes were probed with polyclonal antibodies against p65, MMP-9, epidermal growth factor receptor (EGFR), PKCα, PKCβ, PKCγ, Akt, phosphatidylinositide kinase 3 (PI3K), IκB-α, phosphorylated IκB-α, c-Jun N-terminal kinase (JNK), phosphorylated JNK, p38, phosphorylated p38, extracellular signal-related kinase (ERK), phosphorylated ERK, and β-actin (Cell Signaling Technology, Beverly, MA, USA). Bound antibodies were detected with peroxidase-conjugated anti-rabbit antibodies followed by chemiluminescence (ECL System; Amersham, Buckinghamshire, UK) and autoradiographic exposure. The intensities of gel bands were calculated with a Gel-Pro Analyzer.

### Statistical analysis

One-way analysis of variance (ANOVA) was used to determine whether mean values differed significantly (*p <* 0.05). If the means were significantly different, a Tukey-Kramer *post-hoc* multiple group comparison test was used to compare individual groups. Data are shown as mean ± standard error of the mean (SEM).

## Results

### Gen inhibited TPA-induced invasion and migration in human hepatoma cells

**Figure 1:**
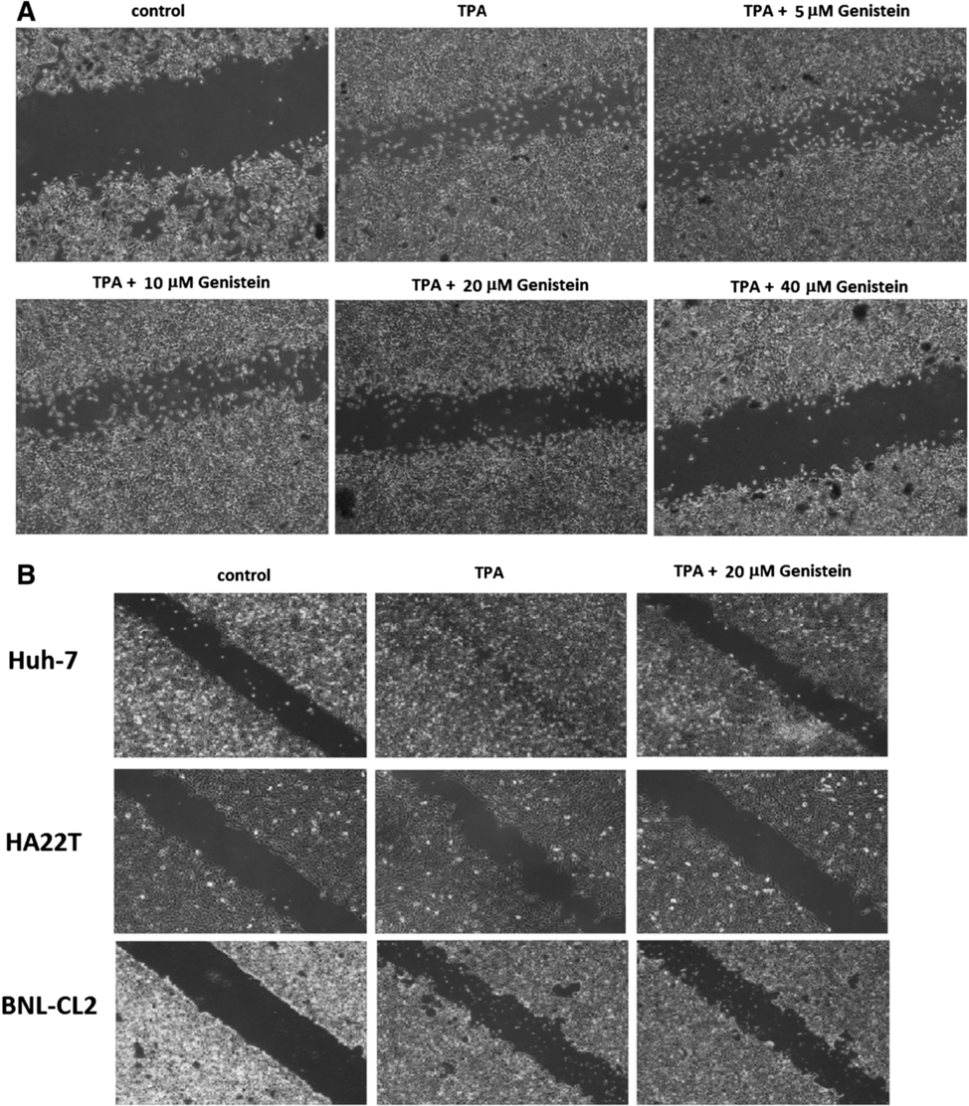
**Effect of genistein (Gen) on TPA-induced migration in hepatoma cell lines.** For the wound healing assay, hepatoma cell lines HepG2 **(A)**, Huh-7, and HA22T and liver cells BNL CL2 **(B)** were treated with 80 μM TPA and different concentrations of Gen in 6-well plates and incubated for 24 h. Photos of the wound were taken using microscopy under 100× magnification.

*In vitro* invasion and migration assays, including transwell and wound healing assays, were used to determine the inhibitory effect of Gen on the invasive potency of human hepatoma cell lines (HepG2, Huh-7, and HA22T) and murine embryonic liver cells (BNL CL2). To determine whether Gen could inhibit TPA-induced migration on the surface of the tissue culture plate, we performed wound healing experiments. As shown in Figure 1A, migration of HepG2 cells was increased by TPA incubation and inhibited by 20 μM and 40 μM of Gen.

HepG2 cells were also treated with TPA and Gen in an invasion chamber to assess the effects of Gen on TPA-induced cell invasion. We also examined the migration of the other 3 cell lines (Figure 1B). The migration of human hepatoma cell lines (Huh-7 and HA22T) was induced by TPA incubation and inhibited by treatment with Gen at 20 μM. However, liver cells (BNL CL2) were not affected by TPA incubation and treatment with Gen at 20 μM.

**Figure 2:**
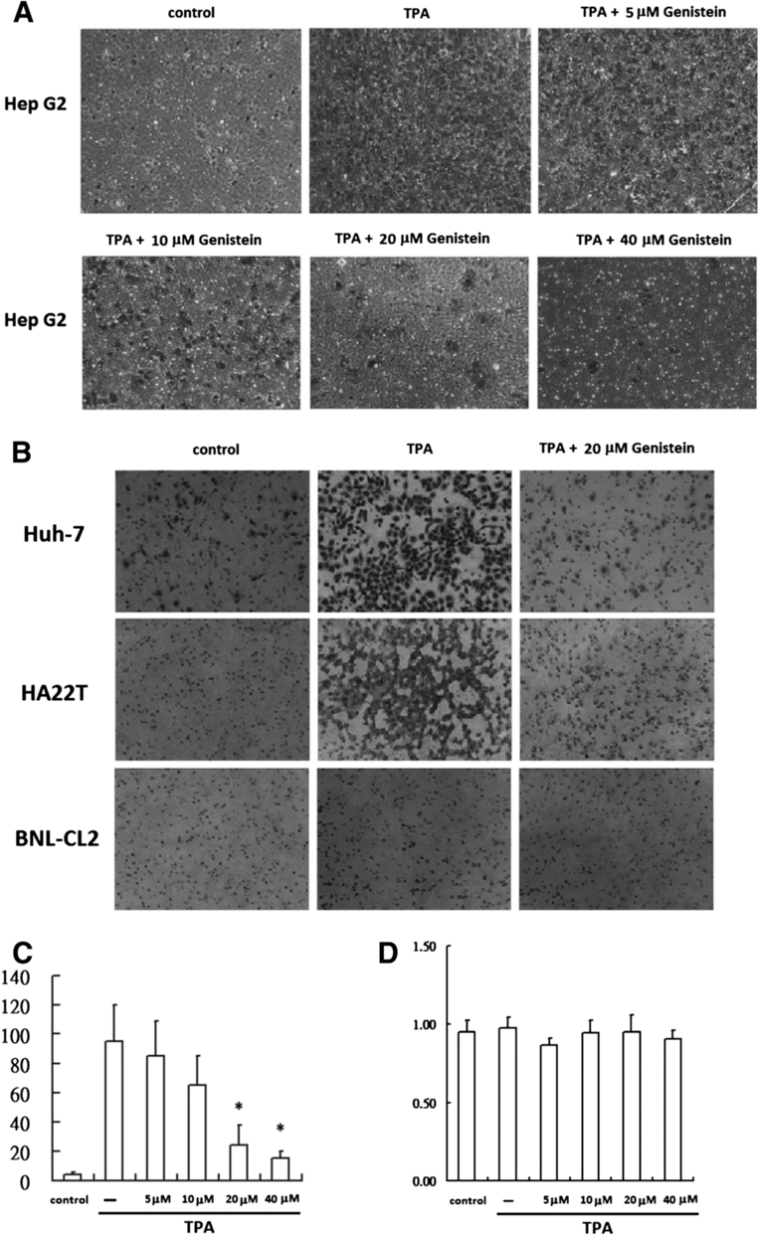
**Effect of genistein (Gen) on TPA-induced cell invasion.** For the invasion assays, hepatoma cell lines HepG2 **(A)**, Huh-7, and HA22T and liver cells BNL CL2 **(B)** (5 × 10^4^) were resuspended in 200 μL of DMEM and added to the upper compartment of Matrigel invasion chambers supplemented with medium and treated with 80 μM TPA with or without different concentrations of Gen. After 24-h incubation, the total number of cells on the lower surface of the insert chambers was stained with crystal violet and counted under a microscope (200× magnification). Quantification of cell invasion assay. HepG2 **(C)**, Huh-7, HA22T cells and liver cells BNL CL2 **(E)** were treated with 5–40 μM or 20 μM of Gen for 24 h. Cell viability of HepG2 **(D)**, Huh-7, HA22T and BNL CL2 cells **(F)** was determined by MTT assay. Results are expressed as fold invasion and presented as the total number of invasive cells in treated cells relative to untreated cells. Values represent the mean ± SEM of 3 independent experiments. *p < 0.01 compared to TPA-treated cells.

We calculated the resulting number of invasive cells. The results of TPA-induced cell invasion from the transwell assays are illustrated in Figure 2A and B. TPA induced a 15 − 20-fold increase in the number of invasive HepG2, Huh7, and HA22T cells that migrated through the Matrigel-coated filters. This phenomenon was significantly inhibited by Gen in a concentration-dependent manner in HepG2 cells (Figure 2C). Quantitative data derived from 3 independent experiments showed that Gen effectively inhibited the invasion of HepG2 cells elicited by TPA (Figure 2C). Similar results were obtained in the other human hepatoma cell lines (Huh-7 and HA22T). However, invasion was not effected by TPA incubation and Gen treatment in the BNL CL2 liver cells (Figure 2B). Taken together, these results showed that Gen inhibited TPA-induced cell motility and transformation, which are essential invasive properties needed for tumor metastasis. As illustrated in Figure 2D, the cytotoxicity of TPA and Gen was evaluated using the MTT assay. No cytotoxic effects were observed for 5–40 μM Gen in HepG2 cells (Figure 2F).

### Effect of Gen on TPA-induced MMP-9 expression and activity

**Figure 3:**
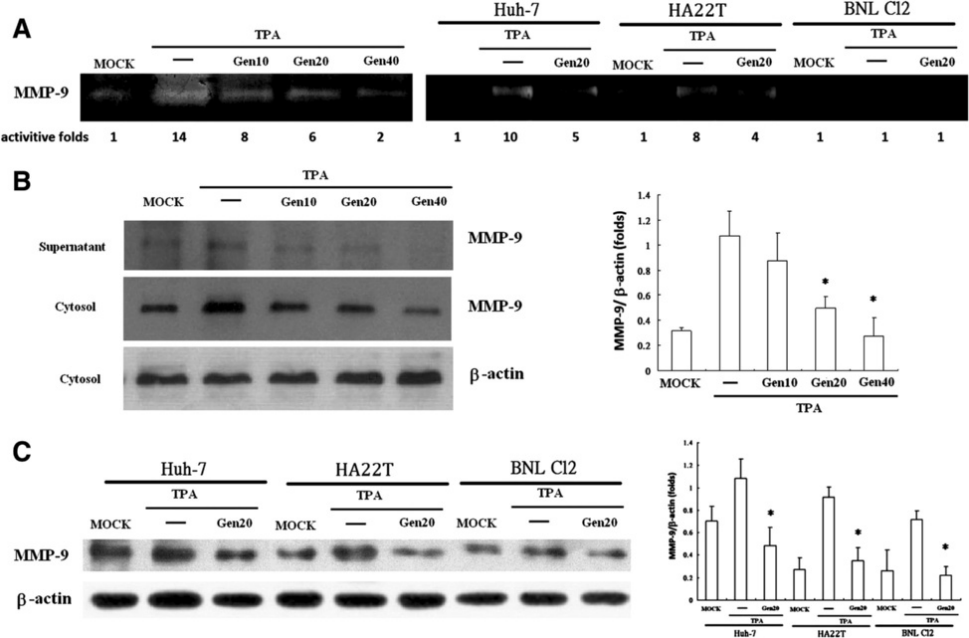
**Effect of genistein (Gen) on TPA-induced MMP-9 expression in hepatoma cell lines.** Conditioned medium was collected from cultures after 24 h and analyzed by gelatin zymography **(A)**. Cells were cultured in 25-cm^2^ flasks, pretreated with 10–40 μM Gen for 1 h, then treated with 80 μM TPA for 16 h in Hep G2 **(B)** and others cell lines **(C)**. Western blot analysis showed MMP-9 secretion into the culture medium (supernatant) and MMP-9 expression in the cytosol. Values represent the mean ± SEM of 3 independent experiments. *p < 0.01 compared to TPA-treated cells.

**Figure 4:**
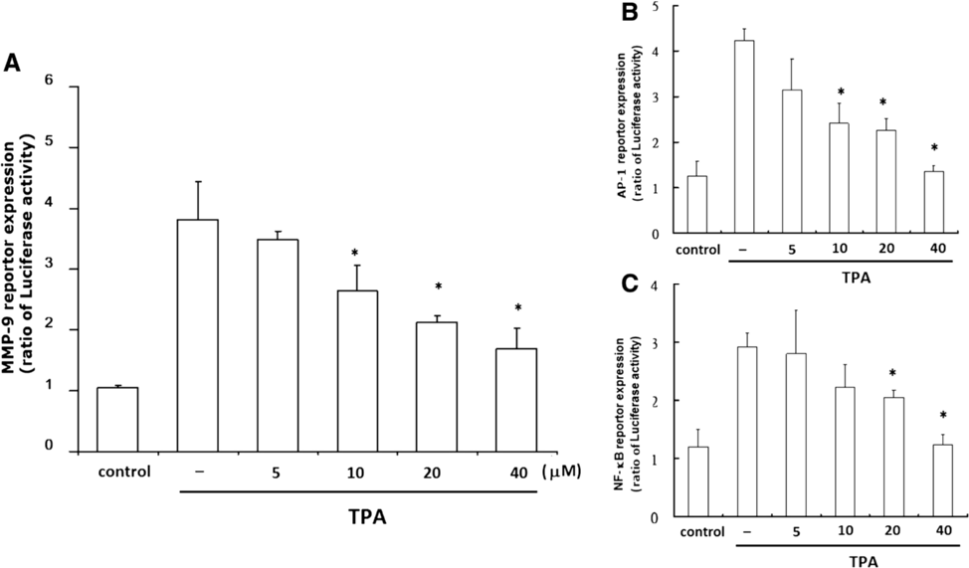
**Effect of genistein (Gen) on TPA-induced *MMP-9*−, *NF-κB*−, and *AP-1*–dependent luciferase reporter gene expression.** HepG2 cells treated with increasing concentrations of Gen were transfected with **(A)***MMP-9*−, **(B)***AP-1*−, or **(C)***NF-κB* containing plasmids linked to the luciferase gene. After 16-h treatment with 80 μM TPA, cell lysates were assayed for luciferase activity. Results are expressed as fold activity over that of untreated transfected cells. Values represent the mean ± SEM of 3 independent experiments. *p < 0.01 compared to TPA-treated cells.

Tumor invasion requires increased expression of MMP-9. To examine whether gelatinolytic MMP activity in hepatoma cells could be activated by TPA, we performed zymographic analysis. Figure 3A shows that treatment with TPA for 24 h significantly increased MMP-9 expression in hepatoma cell lines (HepG2, Huh-7, and HA22T), which was suppressed by Gen. However, the gelatinolytic activity of MMP was not expressed in murine embryonic liver cells (BNL CL2). Gen-mediated suppression of TPA-induced MMP-9 expression resulted from increased MMP-9 levels in the culture medium (supernatant) and cytosol in a concentration-dependent manner (Figure 3B). In addition, 20-μM Gen-mediated suppression of TPA-induced MMP-9 expression resulted from increased MMP-9 concentrations (Figure 3C). Moreover, as shown in Figure 4, Gen dramatically inhibited TPA-induced EGFR expression in Hep G2 cells.

### Effect of Gen on TPA-activated transcription of *MM-9*, *NF-κB,* and *AP-1* promoters

To determine whether the transcriptional activities of *MMP-9*, *NF-κB*, and *AP-1* are regulated by TPA, we examined the promoter activity of the *NF-κB* and *AP-1* genes using luciferase assays. The cells were treated with TPA (with or without Gen) for 16 h, and promoter activity was measured by luciferase assay. Figure 4A shows that the *MMP-9* promoter was increased approximately 4-fold by TPA in HepG2 cells relative to the control *MMP-9* promoter-transfected cells, and the activated promoter was suppressed by Gen in a dose-dependent manner and significantly suppressed at concentrations ≥10 μM. Figure 4B shows that the *AP-1* promoter increased approximately 4-fold over the activity in AP-1–transfected cells in response to TPA, which was also inhibited by Gen in a dose-dependent manner and significantly suppressed at concentrations ≥10 μM. As shown in Figure 4C, the *NF-κB* promoter activity was increased approximately 2.7-fold over that in NF-κB–transfected cells in response to TPA, and this was inhibited by Gen in a dose -dependent manner and significantly suppressed at concentrations ≥20 μM.

**Figure 5:**
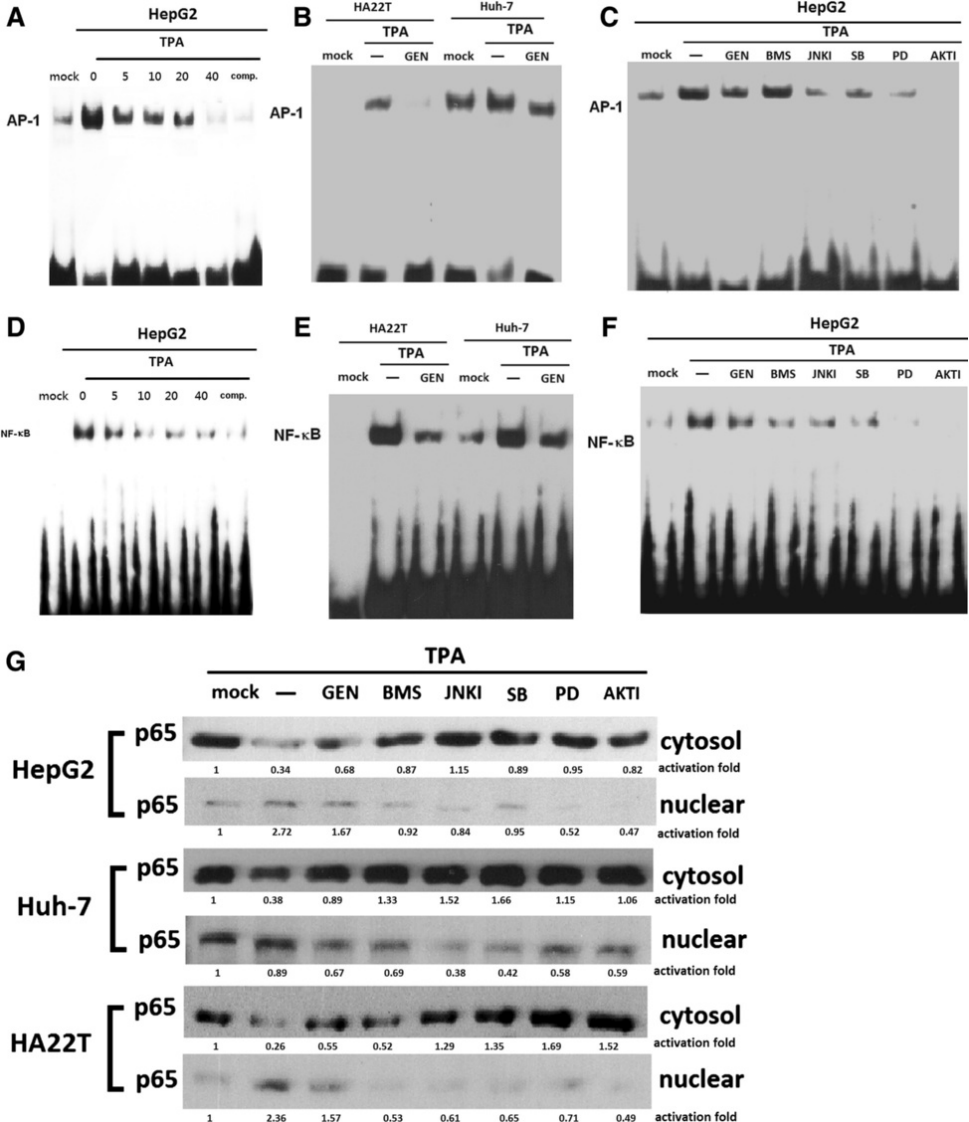
**Effect of genistein (Gen) on TPA-induced NF-κB and AP-1 activation and translocation of p65 in hepatoma cell lines.** After 45 min, EMSAs of nuclear extracts of cells were performed. TPA induced AP-1 **(A, B, C)** and NF-κB **(D, E, F)** activation, which was inhibited by Gen and different inhibitors **(B, E)**. For translocation of p65, cells were pretreated with 20 μM Gen with or without inhibitors for 45 min before incubation with TPA for 45 min. Then, nuclear and cytosolic extracts were prepared and subjected to western blotting with antibodies specific for p65 translocation **(G)**. Cells were pretreated with 20 μM Gen with or without inhibitors for 30 min before incubation with TPA for 45 min, and then nuclear extracts were prepared as described in the Methods section.

To determine whether the inhibitory effect of Gen in TPA-treated cells leads to NF-κB and AP-1 inhibition, the effects of Gen on TPA-stimulated NF-κB– and AP-1–specific DNA-protein binding activity were examined. Biotinylated EMSAs showed that TPA increased DNA-binding of NF-κB and AP-1 after 45 min. Treatment with 20 μM Gen inhibited TPA-induced AP-1–specific DNA-protein binding (Figure 5A and B), and treatment with 20 μM Gen inhibited TPA-induced NF-κB–specific DNA-protein binding compared to TPA-induced cells (Figure 5D and E). We also used specific inhibitors to examine whether TPA-induced DNA-binding of AP-1 and NF-κB. We found that TPA-induced DNA-binding of *AP-1* was decreased by inhibitors of p38 (SB), JNK (JNKI), ERK (PD), and AKT (AKTI) (Figure 5C). Furthermore DNA-binding of NF-κB was decreased by inhibitors of IKK (BMS), JNK (JNKI), and AKT (AKTI) in hepatoma cells (Figure 5F). We also used specific inhibitors to examine the translocation of NF-κB p65. The translocation was aborted by 20 μM Gen and inhibitors of IKK (BMS), JNK (JNKI), and AKT (AKTI) (Figure 5G).

### Inhibitory effect of Gen on TPA-induced activation of MAPKs, PI3K, Akt, and PKC

**Figure 6:**
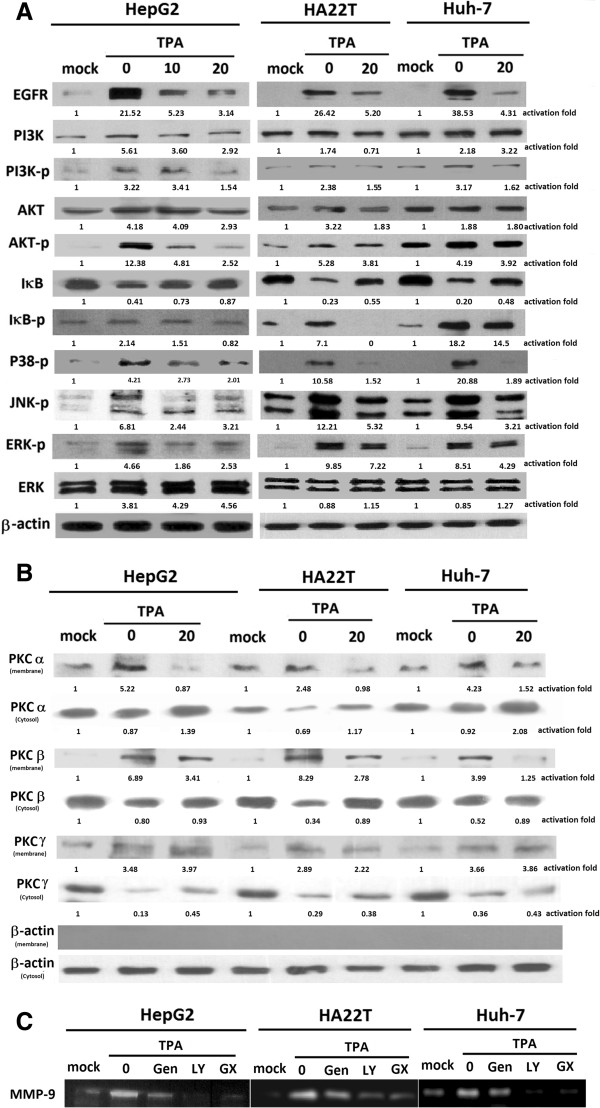
**Effect of genistein (Gen) on TPA-induced EGFR, MAPK, IκB, AKT, PKC-signaling pathways in hepatoma cell lines. (A)** Cells were pretreated with 10 or 20 μM Gen for 30 min before incubation with 80 μM TPA for 45 min or 24 h (for EGFR), then whole-cell lysates were prepared and subjected to western blotting with antibodies specific for phosphorylated PI3k, AKT, IκB, JNK, p38, and ERK as described in Methods section. **(B)** HepG2 cells were cultured in serum-free media containing 80 μM TPA for 45 min, then the cell cytosolic and membrane fractions were prepared and then the extracts were analyzed by western blots as described in Methods section. The values under each lane indicate relative density of the band normalized to β-actin. All analyses were representative of at least three independent experiments. **(C)** The gelatinolytic activities of MMP-9 were determined by gelatin zymography as described in Materials and Methods.

Mitogen-activated protein kinases (MAPKs) are known to regulate AP-1 and NF-κB activation via multiple mechanisms. Studies have shown that the MAPK, IκB, and PI3K/Akt signaling pathways are involved in TPA-mediated induction of EGFR and MMP-9 [26,27]. We investigated the effects of Gen on TPA-induced phosphorylation of ERK, p38, JNK, IκB, and PI3K/Akt activity in hepatoma cells. Western blot analysis revealed that TPA alone caused a significant increase in the phosphorylation of ERK, p38, JNK, IκB, PI3K, and Akt compared to vehicle-treated controls; this phosphorylation was inhibited by pretreatment with Gen (Figure 6A). Our results showed that the TPA-induced phosphorylation of MAPK, IκB, and PI3K/Akt in cells treated with 20 μM Gen was decreased, with the exception of the phosphorylation of p38 and IκB with 20 μM Gen. As illustrated in Figure 6, Gen markedly abrogated TPA-induced MMP-9 enzyme activity through inhibition of the MAPK, IκB, and PI3K/Akt signaling pathways.

## Discussion

Epidemiologic studies have demonstrated that the consumption of fruits and vegetables can reduce the risk of several types of human cancers [28]. Approximately 70% of the drugs used for cancer treatment are derived from or based on natural products [29,30]. A number of phytochemicals can inhibit tumor metastasis and cell invasion via suppression of *MMP* gene expression and enzymatic activity. For example, curcumin interferes with the activity of MMP-9, reducing degradation of the extracellular matrix, which forms the basis of the angiogenic switch [31]. Hesperidin suppresses TPA-induced *MMP-9* transcription by inhibiting NF-κB activity [32], and pterostilbene inhibits TPA-stimulated *NF-κB* and *AP-1* transcriptional activities [27]. Gen is a small, biologically active flavonoid that is abundant in soy. Gen is best known for its ability to inhibit cancer progression and metastasis. Consumption of Gen in the diet has been linked to decreased rates of metastatic cancer in a number of population-based studies [33]. In HCC, Gen has anti-mutagenic activity [34] and induces apoptosis [35]. In the present study, we showed for the first time that Gen suppresses TPA-induced cell invasive activity and MMP-9 expression by reducing tumor migration and invasion of HCC.

Several stimulators increase the expression of MMP-9 via various signaling pathways and result in increased invasiveness in cell lines. Specifically, TPA-induced MMP-9 expression has been studied extensively in HCC cells [14,27,36]. These studies suggest that TPA-induced MMP-9 expression in HepG2 cells occurs by activating phosphorylation of MAPK, IκB, and Akt signaling pathways. These pathways activate the transcription factors NF-κB and AP-1. We previously reported that NF-κB and AP-1 are activated in TPA-induced MMP-9 expression via IκB and MAPK pathways in HCC cells [14]. Another report showed that NF-κB and AP-1 were activated following TPA-induced MMP-9 activation though extracellular signal-regulated MAPK and PI3K/Akt [27,37]. The present study showed that Gen effectively suppressed TPA-induced *MMP-9* gene expression by suppressing the MAPK/AP-1 and PI3K/AKT/NF-κB cascades, with consequent suppression of tumor migration and invasion of human hepatoma HepG2 cells.

EGFR autocrine/paracrine pathways contribute to a number of processes that are important in the development and progression of cancer, including cell proliferation, apoptosis, angiogenesis, and metastatic spread. High expression of EGFR has been observed in numerous human tumors, including lung, colon, breast, head and neck, ovarian, bladder, and liver cancers, and has been shown to correlate with advanced tumor stage and poor clinical prognosis [38-40]. The EGFR signaling pathway is associated with metastatic properties, including cell motility, adhesion, and invasion *in vitro*[41,42]. EGFR activates intracellular signaling cascades, including Ras/Raf/MEK/ERK and PI3K/Akt, and subsequently controls proliferation, migration, and apoptosis [43]. Activation of NF-κB and AP-1 is centrally involved in the induction of the *MMP-9* gene associated with the invasion and metastasis of tumor cells by different agents, including TPA and growth factors, such as EGF [27,44,45]. There is a report that TPA induces EGFR expression in HepG2 cells [27]. Thus, the regulation of NF-κB and AP-1, downstream of the PI3K/Akt and MAPK pathways, might be involved in Gen suppression of TPA-induced MMP-9 expression and invasion in HepG2 cells.

## Conclusion

In conclusion, we provided evidence that Gen promotes anti-invasive and anti-metastatic effects against TPA-mediated metastasis via downregulation of MMP-9 and EGFR and subsequent suppression of NF-κB and AP-1 transcription factors though inhibition of MAPK, IκB, and PI3K/Akt signaling pathways. Therefore, we conclude that MMP-9 inhibitory activity of Gen and its inhibition of multiple signal transduction pathways suggest its therapeutic potential for controlling the invasiveness and metastasis of HCC.

## Abbreviations

Gen: Genistein; MMP-9: Matrix metalloproteinase-9; HCC: Hepatocellular carcinoma; TPA: 12-O-Tetradecanoylphorbol-13-acetate; AP-1: Activator protein-1; NF-κB: Nuclear factor-κB; JNK: c-Jun N-terminal kinase; PI3K: Phosphoinositide 3-kinases; ERK: Extracellular signal-regulated protein kinase; EMSA: Electrophoretic mobility shift assay; MAPK: Mitogen-activated protein kinase; SB: SB203580 P38 inhibitor; PD: PD98059 MEK/ERK inhibitor; JINK: JNK inhibitor; BMS: IKK inhibitor; AKTI: AKT inhibitor; NE: Nuclear extract.

## Competing interests

The authors declare that they have no competing interests.

## Authors’ contributions

CC Yeh, SD Wang, and ST Kao performed the molecular genetic studies, participated in the sequence alignment, and drafted the manuscript. CC Yeh and SD Wang participated in the design of the study and performed the statistical analysis. CJ Liu, ST Kao, and CC Yeh conceived of the study, participated in its design and coordination, and helped to draft the manuscript. All authors read and approved the final manuscript.

## Pre-publication history

The pre-publication history for this paper can be accessed here:

http://www.biomedcentral.com/1472-6882/14/26/prepub
